# TOPS++FATCAT: Fast flexible structural alignment using constraints derived from TOPS+ Strings Model

**DOI:** 10.1186/1471-2105-9-358

**Published:** 2008-08-31

**Authors:** Mallika Veeramalai, Yuzhen Ye, Adam Godzik

**Affiliations:** 1 Joint Center for Molecular Modeling, Burnham Institute for Medical Research, La Jolla, CA 92037, USA; 2 School of Informatics, Indiana University, Bloomington, IN 47408, USA

## Abstract

**Background:**

Protein structure analysis and comparison are major challenges in structural bioinformatics. Despite the existence of many tools and algorithms, very few of them have managed to capture the intuitive understanding of protein structures developed in structural biology, especially in the context of rapid database searches. Such intuitions could help speed up similarity searches and make it easier to understand the results of such analyses.

**Results:**

We developed a TOPS++FATCAT algorithm that uses an intuitive description of the proteins' structures as captured in the popular TOPS diagrams to limit the search space of the aligned fragment pairs (AFPs) in the flexible alignment of protein structures performed by the FATCAT algorithm. The TOPS++FATCAT algorithm is faster than FATCAT by more than an order of magnitude with a minimal cost in classification and alignment accuracy. For beta-rich proteins its accuracy is better than FATCAT, because the TOPS+ strings models contains important information of the parallel and anti-parallel hydrogen-bond patterns between the beta-strand SSEs (Secondary Structural Elements). We show that the TOPS++FATCAT errors, rare as they are, can be clearly linked to oversimplifications of the TOPS diagrams and can be corrected by the development of more precise secondary structure element definitions.

**Software Availability:**

The benchmark analysis results and the compressed archive of the TOPS++FATCAT program for Linux platform can be downloaded from the following web site:

**Conclusion:**

TOPS++FATCAT provides FATCAT accuracy and insights into protein structural changes at a speed comparable to sequence alignments, opening up a possibility of interactive protein structure similarity searches.

## Background

Structural biology is one of the most successful fields of modern biology. Over 50,000 solved protein structures illustrate details of many specific biological processes. The same data also provide us with information about the global features of protein structure space and can be studied to discover the evolutionary, physical, and mathematical rules governing them. How many fundamentally different protein shapes (folds) are there? How do protein structures evolve? How do new structural features appear, and if they are coupled with changes in function, how does this process occur? Such questions can be studied by classifying, comparing and analyzing known protein structures. Two different, but synergistic strategies are typically used for this purpose. In classification systems such as SCOP [1] or CATH [2], human intuition is used to simplify the description of protein structures to a manageable size, and a human eye, sometimes supported by automated analysis, can recognize patterns and types of structures. In the second approach, specialized comparison algorithms, such as DALI [3], CE [4], or FATCAT [5] can be used to calculate a distance-like metric in the protein structure space. This in turn can be used to cluster proteins into groups. Many such algorithms have been developed over the past few decades and have been mostly used for the classification of protein structures into families.

An exact solution of an alignment between two structures is formally equivalent to a threading problem and is therefore NP-complete [6]. However, a practical solution can be obtained by heuristics reducing the problem to a manageable size [7]. In human classification systems, the protein is usually reduced to a set of several structural elements, which obviously involve many arbitrary thresholds. Automated algorithms have the same problem and also suffer from inconsistencies between different numerical measures of protein structure similarity [8]. Interestingly, despite these problems, results of different approaches are broadly similar. They all identify approximately a few hundred general classes of protein structures, usually called folds [1] or topologies [2], distinguished by how the main chain of the protein folds around itself in the three-dimensional space. At the same time, the comparison of different approaches, both between and within the two classes, shows that fold/topologies (or cluster) definitions are somewhat fuzzy, with some proteins being occasionally difficult to classify and joining different groups depending on various assumptions. This lead some to question the concept of the fold [9], but practical application of protein structure comparison leaves little doubt that protein structure space has some natural granularity that overlaps well with the traditional fold classification.

**Figure 1:**
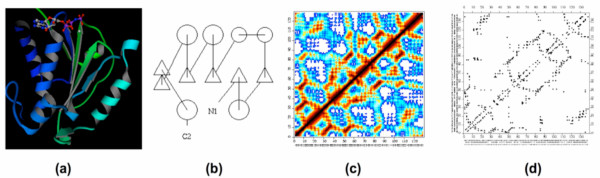
Different representations of the protein structure flavodoxin-fold CheY: (a) ribbon diagram; (b) TOPS style topology diagram; (c) distance; (d) contact map.

Comparison and classification of protein structures is significantly simplified by the fact that proteins have naturally modular structures, being mostly composed of locally regular structures: alpha helices and beta strands. These two types of secondary structures constitute a little over 50% of an average protein's length. With the average length of a secondary structure being around 10 amino acids, this makes it possible to describe protein structure as an arrangement of a much smaller number of elements. Protein structures are often visualized in a simplified form, with the so-called ribbon diagram with secondary structures shown as helices and arrows being the most popular (see Figure 1). This picture can be simplified further by showing individual secondary structure elements as simple symbols (circles or boxes/triangles). These depictions, called fold diagrams, originally proposed in the 70s [10-12] are best captured by a TOPS (Topology of Protein Structures) algorithm, which attempts to automate the process of creation of the topology cartoon [13]. While useful in protein classification, such simplified descriptions are not used in the most popular automated protein structure comparison algorithms such as DALI [3] or CE [4]. Kleywegt and Jones developed a method for finding similar motifs based on comparing distance matrices that are constructed by representing protein as a set of SSEs with their directional vectors and angle between those vectors [14]. Programs that used SSEs either for structure comparison based on hierarchical superposition of both SSEs and atomic representation [15] or for finding common substructures in the comparison process based on subgraph isomorphism, such as [16,17] and recent applications of the TOPS diagram [18,19], usually struggle with translating the comparison results from the secondary structure to the individual residue level. Although the SSM method uses graph-matching procedures at the SSE level followed by an interactive 3D alignment of the protein C-alpha atom [20], it lacks the topological relationships between the SSEs, which are essential features in identifying common scaffolds in distantly related proteins. A TOPS pattern was used to guide the sequence alignment, for instance, to build multiple structural alignments of the distantly related family of beta-rich protein domains [21]. The Multiple Sequence Alignment Tool (MSAT) automates this approach, merging it with a popular ClustalW program [22]. DALI [3], CE [4] or FATCAT [5] introduce their own methods of decomposing the protein structure into smaller units, such as 7 × 7 dense distance map fragments (DALIs) or aligned fragment pairs (AFPs) (CE and FATCAT). The large number of such fragments and the combinations of the fragments that need to be evaluated by structure comparison programs is the main reason for the significant computational requirements of such algorithms. However, more importantly, TOPS+ method is used here to enable a structural comparison that takes into account flexibility in protein structures and not only classifies the differences, but also can recognize such rearrangements – which is a first such application using the SSEs language. In this contribution, we explore the question of whether it would be possible to combine insights provided by topology diagrams into automated protein structure alignment algorithms, focusing on the FATCAT program developed previously in our group.

## Methods

### Flexible structure alignment method FATCAT

**Figure 2:**
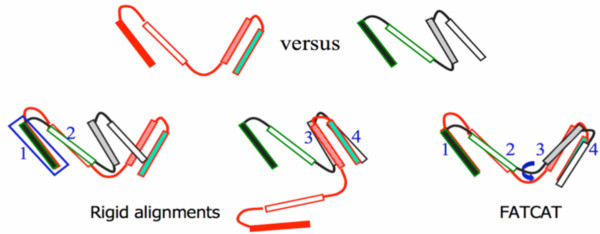
Rigid versus flexible alignment of aligned fragment pairs (AFPs).

FATCAT [5] is a unique structure alignment method that allows for flexibility in the structures being compared. It builds the alignment by chaining aligned fragment pairs (AFPs) [23] together using a unified scoring function where AFP extensions, gaps, and twists each have their specific scores (Figure 2). Introducing a twist into the alignment is penalized, but this penalty may be compensated for by the gain in the score of the resulting alignment (i.e., longer alignment and/or better RMSD). Rigid alignment can be treated as a special case, in which no twist is allowed in chaining AFPs. FATCAT program provides alignment in both, "rigid" mode and "flexible" (default) mode.

FATCAT, as well as most other protein structure comparison programs, is very slow when compared to sequence alignments. The computing time of FATCAT is determined by the size of the collection of AFPs detected between the two structures being compared. FATCAT is available from a server  with an option to search in SCOP or PDB databases for similar structures. This search typically takes between 8 to 16 hours of CPU time, and this is the main obstacle to broader use of this option. FATCAT has been used to construct a Flexible Structure Neighborhood (FSN) database that contains pre-computed results of structure similarity searches and it takes several weeks of CPU time to update the FSN database. Other protein structure comparison resources, such as DALI or CE have very similar problems.

### TOPS cartoons and TOPS graph models

As discussed in the Background, TOPS cartoons capture the simplified, fold-level description of protein structure and at the same time can be automated [24]. The TOPS algorithm uses structural features such as hydrogen bonds and chirality of the beta strands to provide a scoring function to optimize the cartoon (see Figure 1(b)). In TOPS, the secondary structural elements (SSEs) are derived from the DSSP program [25]. Based on TOPS cartoons, a formal graph model and graph-based definitions of protein topology and pattern discovery and comparison methods were developed [26,27]. The TOPS database and comparison, pattern discovery and matching programs are accessible from .

### Novel TOPS+ and TOPS+ strings models

**Figure 3:**
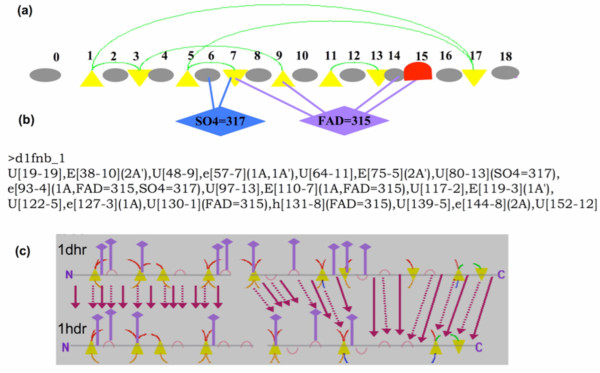
**(a) TOPS+ graph model, (b) TOPS+ strings model, and (c) TOPS+ strings matches between *Dihydropteridine reductase *from rat (*1dhr*) and human (*1hdr*).** All the conserved TOPS+ strings elements are shown with pink arrows. Dotted arrows indicate matched helices and strands, plain arrows indicate matched loops, and arrows with double lines indicate matched ligand-interacting loops.

The TOPS model was further enhanced to incorporate features such as protein-ligand interaction information and more detailed secondary structural segment information. This enhanced model is called TOPS+ model (see Figure 3a). This TOPS+ model can be described formally in a TOPS+ strings language (Figure 3b) at a reduced linear level. The enhanced TOPS+ strings models can be used in fast string-based structure matching and comparison, at the same time avoiding issues of NP-completeness associated with graph alignments.

In detail, each node (SSE segment) of the TOPS+ strings is described by its type, orientation, PDB start number, segment length, total number of incoming (InArc) and outgoing (OutArc) arcs (edges), total number of ArcTypes, and total number of ligand arcs (LigArc). The type of the segment (SSEType) could be one of [E, e, H, h, U, u], where, "E" and "e" represent the "up"- and "down"-oriented beta strands; "H" and "h" indicate the "up"- and "down"-oriented alpha helices; and "U" and "u" represent ligand-bound and ligand-free loops. The InArcType can be classified as an/a [R, L, P, A], where "R" and "L" represent right and left chiralities; and "P" and "A" represent parallel and anti-parallel hydrogen bonds, respectively. The OutArcType is represented in a similar manner by [R', L', P', A']. Ligand arcs are indicated by LT = AA, where LT is the ligand type and AA is the PDB number. For example, Figure 3(a) and 3(b) contain visual representations of TOPS+ and TOPS+ strings models, respectively, for the protein domain *d1fnb_1*. Here the triangles represent the beta strands; the red curve represents the alpha helix; gray ellipsoids indicate loops; and green arcs indicate hydrogen bonds between two beta strands, called anti-parallel beta sheets. The length of a TOPS+ strings model is defined by number of SSEs; thus, the length of *d1fnb_1 *is 19. For further details, see [28].

### TOPS+ strings comparison method

TOPS+ is a comparison method that computes a distance between TOPS+ strings models of two proteins based on a dynamic programming approach and identifies the longest common subsequence (LCS), consisting of the list of the topologically equivalent SSEs between two proteins. For example, Figure 3(c) shows the TOPS+ strings alignment between Dihydropteridine reductase proteins from rat (1dhr) and human (1hdr). The TOPS+ strings models for 1dhr and 1hdr are represented by a linear string-model, where a yellow triangle and red curves indicate the beta strands and alpha helices in their "up" or "down" orientations, respectively. The grey line and purple stubs represent the loop regions and the NAD ligand interactions, respectively. Note that the ligand-interaction information is optional and in this work we have not used it. The incoming and outgoing arcs are depicted in the SSEs (top and bottom of the beta strands), where red and green arcs represent the parallel and anti-parallel hydrogen-bond interactions that show beta-sheet information, while yellow and blue arcs indicate the right and left chirality relationships between the SSEs. A pink arrow between the TOPS+ strings elements indicates the conserved SSE. The dotted arrows indicate the conserved alpha helices and beta strands, while the plain arrows indicate the conserved loop regions.

### TOPS++FATCAT method

**Figure 4:**
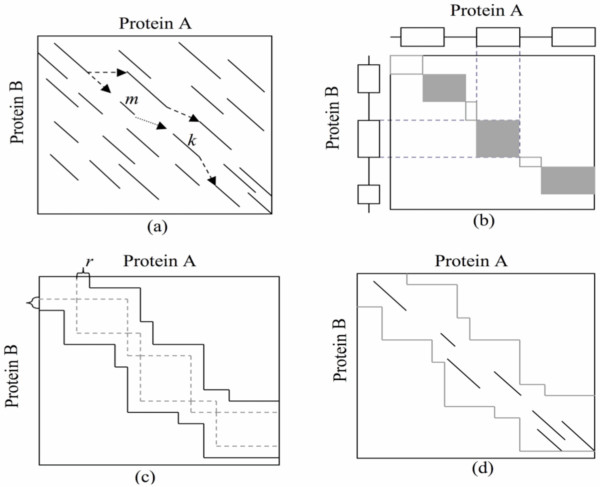
**The schematic illustration of FATCAT structural alignment by chaining AFPs in a constrained alignment region defined by TOPS alignment output.** (a) In FATCAT, two fragments form an AFP (shown as a line in the graph) according to the criteria (see text). (b) The alignment of secondary structure elements from TOPS+ comparison is used to define the constrained area for AFP detection, in which each two aligned secondary structure elements defines an "eligible" block (shown as filled squares). These blocks may be disconnected, and we need to connect them with connecting blocks (shown as open squares). (c) We add a buffer area surrounding the constrained area defined in (b) (shown as the area closed by dashed lines) to get the *constrained alignment region *for FATCAT alignment (show as the area closed by dark lines). (d) Only those AFPs within the constrained alignment region are used in the dynamic programming algorithm for chaining.

In this work, we want to test the general idea of pruning the search space of the FATCAT comparison process using topological constraints derived from the TOPS+ strings alignment. Many of the AFPs considered in the FATCAT alignment could be easily eliminated from the comparison by constraining the alignment region. Here we explore constraints obtained from the TOPS+ strings alignment, which identifies topologically equivalent secondary structure elements (alpha helices, beta strands, and loops) for this purpose. Such equivalences define blocks that restrict the alignment region; AFPs that fall outside these regions are simply not considered (see Figure 4(b)). We introduce a parameter *r *to control the strictness of constraints by TOPS+ strings alignments; *r *equals 0 if the alignment region is strictly restrained by TOPS+ strings alignment, and *r *is set to 1 by default in our program to allow certain flexibility to the constrained alignment region (Figure 4(c)). We then can speed up the FATCAT alignment by considering only the AFPs within the constrained alignment area (Figure 4(d)). The rigid structural alignment can be treated as a special case of TOPS++FATCAT, in which no twist is allowed in chaining AFPs. However, the TOPS++FATCAT program provides alignment in both, "rigid" mode and "flexible" mode (default).

### Benchmarking

For benchmarking and comparison, we have used the PDB40 dataset of 1,901 protein domain pairs (DP) corresponding to SCOP version 1.61 from the ASTRAL database [29]. Table 1 provides the SCOP superfamily level homolog versus non-homolog statistics for the four main SCOP classes i.e., *all-alpha*, *all-beta*, *alpha/beta*, *alpha+beta*, and all proteins regardless of their structural classes.

**Table 1:** SCOP Superfamily-Level Homolog vs Non-Homolog Protein Domain Pairs Statistics

SCOP Domains	Protein Domains from same superfamily (Homolog)	Protein Domains from different superfamily (Non-Homolog)	Total Number of Domain Pairs
All alpha Class	90	18	108
All beta Class	95	42	137
Alpha/beta Class	226	200	426
Alpha+beta Class	93	71	164
All Proteins	568	1,333	1,901

### Evaluation Analyses

We performed the Receiver Operating Characteristics (ROC) curve and the AUC (Area Under the ROC Curve) analyses to compare the performance of the TOPS++FATCAT method with the original FATCAT method, using SCOP classification at the superfamily level as a standard of comparison [30].

## Results

### ROC and AUC Analyses

**Figure 5:**
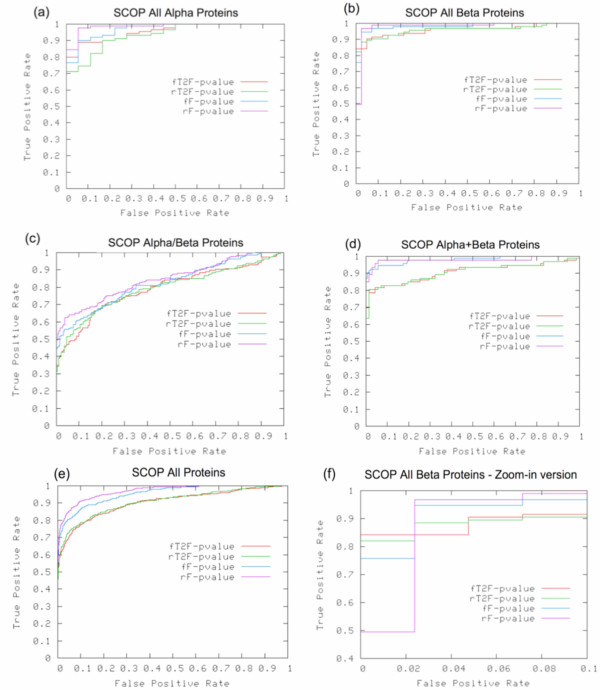
The ROC curve analysis results based on P-values obtained from flexible and rigid options from the FATCAT and TOPS++FATCAT methods, where rF-pvalue and fF-pvalue indicate rigid and flexible FATCAT methods, respectively; similarly, rT2F-pavlue and fT2F-pvalue represents rigid and flexible TOPS++FATCAT methods, respectively.

We have compared the performance of the TOPS++FATCAT method against the original FATCAT method using the SCOP classification information at the superfamily level. We have plotted the ROC curves based on P-values obtained from the FATCAT and the TOPS++FATCAT methods. We have plotted the ROC curves separately for the main SCOP classes, i.e., *all-alpha*, *all-beta*, *alpha/beta*, *alpha+beta*, and all proteins regardless of the class they belong to (see Figure 5(a) to 5(e)). In the graph, the *x- *and *y*-axes represent the false positive and true positive rates of the performance of the comparison methods respectively. In the legend, rF-pvalue and fF-pvalue indicate results from the *rigid *and *flexible *FATCAT methods, respectively; similarly, rT2F-pvalue and fT2F-pvalue represent the *rigid *and *flexible *TOPS++FATCAT methods, respectively. We have calculated the AUC values for all the SCOP classes based on ROC curves obtained from the FATCAT and TOPS++FATCAT methods with the flexible/rigid options (see Table 2).

**Table 2:** AUC Values Based on p-values from the FATCAT and TOPS++FATCAT Methods.

SCOP Domains	FATCAT (Flexible)	TOPS++FATCAT (Flexible)	FATCAT (Rigid)	TOPS++FATCAT (Rigid)
All Alpha Class	95	93	96	91
All Beta Class	97	95	97	95
Alpha/Beta Class	82	79	84	79
Alpha+Beta Class	98	91	97	91
All Proteins	95	90	97	91

For all protein classes, the rigid FATCAT performs best, usually followed by the flexible FATCAT, the rigid TOPS++FATCAT, and the flexible TOPS++FATCAT. The performance of all four methods is best for all alpha and all beta proteins, and all four perform markedly worse (but similar to each other) for alpha/beta proteins. Only alpha+beta proteins show a clear difference between the FATCAT and TOPS++FATCAT methods. It is important to note that the TOPS+ strings models consider the parallel and anti-parallel properties of the beta-sheet information in the form of total number of incoming and outgoing arcs with their ArcTypes. Thus, the TOPS++FATCAT method discriminates the protein domain pairs more efficiently compared to the original FATCAT method. For example, in the all-beta protein domain pairs, both the flexible and the rigid TOPS++FATCAT methods perform well. The flexible TOPS++FATCAT method covers nearly 84% of protein domains with 0% false positives, but the flexible and rigid FATCAT methods cover only 76% and 49% of the true positives, respectively, with 0% false positives. The zoomed-in version of the ROC curves with up to 10% false positives for all-beta rich protein families is shown in Figure 5(f); where both the rigid TOPS++FATCAT (green) and flexible (red) TOPS++FATCAT methods have coverage rates of 82% and 84% true positives respectively with 0% false positives. The overall results for all protein classes show that TOPS++FATCAT performance is only slightly lower (3%–7% AUC value difference (see Table 2)) as compared to FATCAT while providing a significant, more than 10-fold speedup (see next section).

### AFP and Runtime Analyses

**Figure 6:**
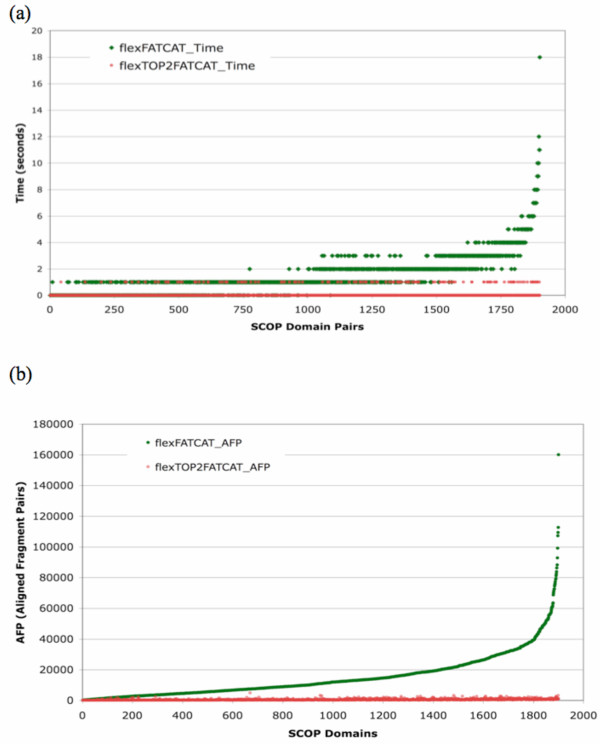
Graph showing the runtime and AFP analysis of the FATCAT (in green) and TOPS++FATCAT (in red) methods based on the flexible option, (a) runtime statistics, where the *x*-axis indicates the 1,901 SCOP domain pairs ordered by flexible_FATCAT runtime; (b) total number of AFP statistics, where the *x*-axis represents the 1,901 SCOP domain pairs ordered based on AFPs from the flexible_FATCAT method.

We tested both the FATCAT and TOPS++FATCAT methods using the Mac OS X version 10.4.10 computer system with a 2 × 2.66-GHz Dual-Core Intel Xeon processor and 1-GB 667 MHz memory. We have performed runtime analysis on 1,901 protein domain pairs and counted the total number of AFPs and the corresponding runtime from both the FATCAT and the TOPS++FATCAT methods. The results show an exponential increase in AFPs (Figure 6(b)) and corresponding runtime (Figure 6(a)) for the FATCAT method as compared to the TOPS++FATCAT method (see Table 3) For example, the average number of AFPs for the TOPS++FATCAT method is 530, but the average number of AFPs for the FATCAT method is 15,019. This represents the number of average AFPs used by the FATCAT method is increased by a factor of 28 (see Table 3). This result leads to the conclusion that TOPS++FATCAT is 22 times faster compared to the FATCAT because this method must take into account more number of AFPs in the comparison process (see Table 3).

**Table 3:** AFP and Runtime from FATCAT and TOPS++FATCAT.

Methods	AFPs (total)	AFPs (average)	Runtime (sec)	Average Runtime (sec)
flexible_FATCAT	28,550,682	15,019	2,738	1.44029
flexible_TOPS++FATCAT	1,007,601	530	122	0.06417
rigid_FATCAT	28,550,682	15,019	2,743	1.44292
rigid_TOPS++FATCAT	1,007,601	530	122	0.06417

### Case Studies

**Figure 7:**
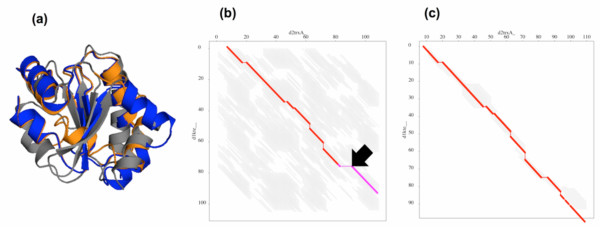
(a) Superposition of d2trxa_(gray) and d1kte__(orange) from flexible_FATCAT and d1kte__(blue) from flexible_TOPS++FATCAT; (b) AFP chaining alignment from flexible_FATCAT; (c) AFP chaining alignment from flexible_TOPS++FATCAT.

While the overall accuracy of both rigid and flexible FATCAT methods is better than their TOPS++FATCAT equivalents, an interesting example where the opposite is true lies in the comparison of two proteins, d2trxa_ (108 aa) from *Escherichia coli *and d1kte__ (105 aa) from *Sus scrofa *(pig) from the thioredoxin-like superfamily. For this pair, the flexible_TOPS++FATCAT method provides an alignment with 88 equivalent positions with 1.67 Å chain RMSD and 3.06 Å of optimal RMSD without any twist, giving the alignment with 10% sequence identity (see Table 4). On the other hand, the flexible_FATCAT method provides an alignment with 86 aligned positions using a twist in the C-terminal region; it has a higher chain RMSD of 5.14 Å, and its optimal RMSD is 3.48 Å. For more information regarding the chain and optimal RMSDs refer [5]. The flexible_FATCAT method uses the twist to align a helix in the C-terminal region, which is positioned incorrectly with a beta-sheet core (see Table 4). Figure 7(a) shows the superposition of d2trxa_ (gray) and d1kte__ (orange) domains from the flexible_FATCAT method, where the blue color indicates the d1kte__ protein domain from the flexible_TOPS++FATCAT method. The incorrect alignment of the C-terminal domain alpha helix of the d1kte__ domain (orange) is visible in the core of the beta-sheet region. Figure 7(b) and 7(c) shows the AFPs from the flexible_FATCAT and flexible_TOPS++FATCAT methods, respectively. The hinge region provides a twist in the flexible_FATCAT method indicated by an arrow and the AFPs represented by a different color (see Figure 7(b)). In this case, the alignment constraints from the TOPS+ strings alignment allow the TOPS++FATCAT method to avoid a spurious alignment.

**Table 4:** Flexible and rigid FATCAT and TOPS++FATCAT comparison results for d2trxa_ and d1kte_

Methods	Optimal RMSD (Å)	Chain RMSD (Å)	Optimal length	Alignment length/gaps	Score	P-value	Sequence Identity (%)	Sequence Similarity (%)	AFPs
Flexible_FATCAT	3.48	5.14	86	107/21	125.51	3.29e-03	9.35	25.23	3,092
rigid_FATCAT	3.06	3.14	88	109/21	123.09	7.14e-5	10.09	25.69	3,092
flexible_TOPS++FATCAT	3.06	1.67	88	109/21	110.05	2.08e-04	10.09	25.69	323
rigid_TOPS++FATCAT	3.06	1.67	88	109/21	110.05	2.08e-04	10.09	25.69	323

**Figure 8:**
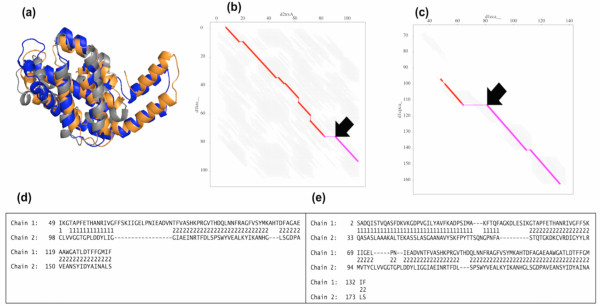
(a) Superposition of d1eca__ (gray) and d1cpca_ (orange) from flexible_FATCAT and d1cpca__ (blue) from flexible_TOPS++FATCAT; (b) AFP chaining alignment from flexible_FATCAT; (c) AFP chaining alignment from flexible_TOPS++FATCAT; (d) structural alignment from flexible_TOPS++FATCAT; (e) structural alignment from flexible_FATCAT.

Optimal RMSD: The root mean square deviation (RMSD) of aligned Cα atoms of the input structures, with one input structure rearranged if flexibility is detected (i.e., twists are introduced in the alignment). Chain RMSD: The RMSD of aligned Cα atoms of the input structures, *without *structural rearrangement even if structural flexibility is detected in the alignment. Optimal Length: The number of equivalent positions of the alignment; P-value. P-value is the probability of observing a greater score used in FATCAT to evaluate the significance of structural similarity detected by FATCAT. AFPs: Aligned Fragment Pairs. For further information, please refer to the FATCAT help page available at the following web site:

The Erythrocruorin protein domain d1eca__ (136 aa) from *Chironomus thummi *and the Phycocyanin alpha subunit protein domain d1cpca_ (162 aa) from *Fremyella diplosiphon *(Cyanobacterium) belong to the Globin-like superfamily. For these protein domain pairs, the FATCAT method provides a better alignment with 120 and 118 aligned positions with the chain RMSD of 4.02 Å based on the flexible and rigid options, respectively. The flexible_TOPS++FATCAT method gives an alignment of 63 aligned positions with the 3.23 Å optimal RMSD and the 6.28 Å chain RMSD. In this case, the flexible_TOPS++FATCAT method misses the N-terminal region helix and misaligns some helices. For example, Figure 8(a) shows the superposition of d1eca__ (gray) and d1cpca_ (orange) domains from the flexible_FATCAT method, while d1cpca_ (blue) domain is from the flexible_TOPS++FATCAT method. The AFP chaining alignment and the actual alignment from FATCAT are shown in Figure 8(b) and 8(e), respectively. Figure 8(c) shows the AFP alignment from TOPS++FATCAT, in which this method misses the N-terminal region and incorrectly aligns some of the C-terminal regions (see Figure 8(d)). However, the rigid_TOPS++FATCAT method produces an alignment of 108 aligned positions with optimal and chain RMSDs of 3.22 Å and 6.28 Å respectively. In general, TOPS comparison does not work well for alpha-rich proteins due to the lack of hydrogen bonds between SSEs [26]. The same is true for TOPS+ strings comparison to some extent; however, this method takes advantage of ligand-interaction information to compare protein domains more efficiently; for example the DNA binding motifs such as *helix-turn*-*helix *and *helix-loop-helix *can be easily recognized [28]. However, we have not explored that ligand pattern discovery option within the TOPS+ strings comparison in this paper. In addition, the TOPS+ strings alignment provides only a basic alignment; the scoring function to find the best alignment has not been optimized. These problems can be addressed in future development by considering the advanced TOPS+ and TOPS+ strings models based on helix-helix packing relationships and SSE-ligand interaction properties together with the *right *and *left *chiralities. Furthermore, the TOPS+ strings comparison can be optimized in both the comparison process as well as in the alignment process in order to take into account *indels *(insertion/deletion) of SSEs which exist in nature across the different members of the protein superfamilies [31].

## Discussion and conclusion

The overall results for all protein classes show that TOPS++FATCAT performance is only slightly lower (3%–7% AUC value difference) as compared to FATCAT while providing a significant, more than 10-fold speedup. The main reason for the discrepancies is that TOPS+ strings alignments occasionally misalign the secondary structure elements and subsequent FATCAT alignment, constrained by the TOPS+ strings alignment, cannot overcome the earlier errors. There is a clear trade-off between the runtime and the accuracy; limiting the pool of fragments being compared speeds up the algorithm but results in (slightly) lower accuracy. At the same time, these results offer clear suggestions for future development. Using a more advanced version of the TOPS+ strings comparison method would remove some of the false positives might be at a cost of significantly slowing the total performance of the TOPS++FATCAT method.

## Authors' contributions

MV developed the TOPS++FATCAT algorithm, performed the calculations and prepared the figures, YY provided advice and oversight in the project, verified the code and provided FATCAT results for comparison, AG contributed to the original idea and to writing of the manuscript.
